# A physically active lifestyle is associated with lower long-term incidence of bipolar disorder in a population-based, large-scale study

**DOI:** 10.1186/s40345-022-00272-6

**Published:** 2022-11-01

**Authors:** Martina Svensson, Sophie Erhardt, Ulf Hållmarker, Stefan James, Tomas Deierborg

**Affiliations:** 1 Experimental Neuroinflammation Laboratory, Department of Experimental Medical Science, Lund University, BMC B11, 221 84 Lund, Sweden; 2 Department of Physiology and Pharmacology, Karolinska Institute, Stockholm, Sweden; 3 Department of Medical Sciences, Cardiology, Uppsala University, Uppsala, Sweden; 4 Department of Internal Medicine, Mora Hospital, Mora, Sweden

**Keywords:** Exercise, Psychiatric disorder, Mental health, Women, Men, Long-term effect

## Abstract

**Background:**

Physical activity has been proposed to be beneficial for the symptomatic control of bipolar disorder, but the duration of the effects, sex-specific mechanisms, and impact of exercise intensity are not known.

**Method:**

With an observational study design, we followed skiers and age and sex-matched non-skiers from the general population to investigate if participation in a long-distance cross-country ski race (Vasaloppet) was associated with a lower risk of getting diagnosed with bipolar disorder. Using the Swedish population and patient registries, skiers in Vasaloppet and age and sex-matched non-skiers from the general population were analyzed for any diagnosis of bipolar disorder after participation in the race. Additionally, we used finishing time of the ski race as a proxy for intensity levels to investigate if exercise intensity impacts the risk of bipolar disorder among the physically active skiers.

**Results:**

Previous participation in a long distance ski race (n = 197,685, median age 36 years, 38% women) was associated with a lower incidence of newly diagnosed bipolar compared to an age and sex-matched general population (n = 197,684) during the up to 21 years follow-up (adjusted hazard ratio, HR = 0.48). The finishing time of the race did not significantly impact the risk of bipolar disorder in men. Among women, high performance (measured as the finishing time to complete the race, a proxy for higher exercise dose) was associated with an increased risk of bipolar disorder compared to slower skiing women (HR = 2.07).

**Conclusions:**

Our results confirm that a physically active lifestyle is associated with a lower risk of developing bipolar disorder. Yet, to elucidate the direction of causality in this relationship requires complementary study designs. And the influence of physical performance level on the risk of bipolar disorder warrants further examinations among women.

**Supplementary Information:**

The online version contains supplementary material available at 10.1186/s40345-022-00272-6.

## Background

With mood swings ranging from severely depressed to overactive mania, bipolar disorder is a complicated diagnosis. Around 2–4% of the population is estimated to have this disorder (NIH, Mental Health). Due to its early onset, chronicity, and severity (Ferrari et al. 2016; Grande et al. 2016), the disability caused is significant. Bipolar disorder is distinguished from unipolar depression by its periodicity and fluctuation in mood and energy states, typically displaying reoccurring depressive episodes and at least one episode of mania or hypomania. Mixed episodes, including both manic and depressive symptoms without recovery in between is also characteristic of bipolar disorder. Still, since the disorder typically begins with one or several depressive episodes, a substantial number of patients are first misdiagnosed with unipolar depression (Daveney et al. 2019; Tondo et al. 2014). As opposed to unipolar depression, bipolar disorder affects both sexes with around the same prevalence, but the frequency of certain symptoms may differ between men and women (Diflorio and Jones 2010; Arnold 2003; Parker et al. 2014; Azorin et al. 2013). For instance, manic episodes and co-occurrence of substance abuse seem to be more common in affected men, whereas depressive episodes and comorbidity with other psychiatric diagnoses, such as anxiety, tend to be more common in women with bipolar disorder (Kawa et al. 2005; Arnold 2003). Nonetheless, around 10 years shorter life expectancy is seen for both men and women diagnosed with bipolar disorder compared to the general population (Kessing et al. 2015a). This reduction is likely attributable to poor cardiovascular health and other somatic comorbidities as well as an increased risk of suicide among these patients (Lomholt et al. 2019; Kessing et al. 2015b; Dome et al. 2019).

The interest is growing in whether physical activity can prevent vulnerable individuals from developing bipolar disorder or improve coping with the disorder if affected. Existing scientific evidence regarding this is scarce (Stubbs et al. 2018) and recommendations are mainly based on extrapolation from what is known about unipolar depression (Thomson et al. 2015). This may be problematic since the manic features of bipolar disorder in many ways reflects an opposite pattern to that of depression. In general, it has been shown that patients with bipolar disorder are significantly less physically active and fit compared to the general population (Vancampfort et al. 2017a, 2017b, 2016; Shah et al. 2007; Pereira et al. 2019). However, the physical activity levels appear to differ substantially between patients in a current depressive state and those in current mania/hypomania (Sylvia et al. 2013; Grunerbl et al. 2015), usually showing high activity levels associated with manic episodes (Melo et al. 2016; Sylvia et al. 2013; Kang et al. 2018) and lower levels with depressive episodes (Sylvia et al. 2013; Beiwinkel et al. 2016). Some studies even suggest that high levels of physical activity are linked to an increased risk of bipolar disorder or mania (Alosaimi et al. 2018; Strohle et al. 2007; Dakwar et al. 2012), leading to speculation on whether vigorous exercise can trigger mania. However, many of these association studies are cross-sectional, making it impossible to investigate the causal direction of these relationships (Sylvia et al. 2013).

So far, the only large-scale prospective study on the association between physical fitness and risk of developing bipolar disorder is the study published by Åberg et al. following over a million Swedish men for up to 40 years (Aberg et al. 2012). Interestingly, they found the lowest fitness levels to be associated with a higher risk of getting bipolar disorder, whereas having a median fitness level was associated with a lower risk of developing bipolar disorder when compared with the highest fitness level, thus indicating that the level of fitness or dose of physical activity also matters. Nonetheless, only men were included in this study and we recently found that physical performance level may impact the risk of developing other psychiatric disorders differently among men and women (Svensson et al. 2019, 2021).

The aim of this study is to examine the association between a physically active lifestyle and the development of bipolar disorder in men and women separately using a population-based cohort with a long-term perspective. In addition, we investigate the impact of fitness level as a proxy for exercise dose on the risk of developing bipolar disorder. We compared participants in the world's largest long-distance cross-country ski race (Vasaloppet) with matched non-skiers from the general population, including a total of 395,369 individuals with up to 21 years of follow-up. To our knowledge, the association between a physically active lifestyle and the development of bipolar disorder has not previously been investigated in a large study population including both men and women with a long follow-up time.

## Methods

### Study design

Our study design has been approved by the Ethical Review Board in Uppsala, Sweden, (D.nr 2010/305) as previously described (Svensson et al. 2019). The study population includes all Swedes who participated in the world’s largest long-distance (30 to 90 km), cross-country ski race (Vasaloppet) between 1989 and 2010 (n = 197,685), as well as frequency-matched, individuals from the general population (n = 197,684) (Additional file 1: Fig. S1). We used Statistics Sweden for frequency matching by taking non-skier controls from the population registry according to sex, region of residency, age group (5 year intervals), and year of participation in ski race as described previously (Hallmarker et al. 2018). On average, Vasaloppet skiers smoke less and have higher leisure-time physical activity, a healthier diet, and lower mortality compared to the general Swedish population (Farahmand et al. 2003; Carlsson et al. 2007). To reduce bias due to the inability to participate in the race because of poor physical health, we excluded individuals with severe disease as previously described (e.g. cancer, chronic neurologic disease, dementia, heart-and lung disease) (Hallmarker et al. 2016). Additionally, we excluded participants with dementia [all-cause, Alzheimer´s disease (AD), vascular dementia (VaD), Parkinson disease dementia, Lewy body dementia, senile dementia], Parkinson disease, meningitis/encephalitis, epilepsy, psychiatric disorders (depressive episode, schizophrenia, bipolar disorder, anxiety disorders and mental disorders due to the use of alcohol) at baseline (see Additional file 1: Table S1).

We additionally monitored skiers for finishing time in the race in three categories with finishing times of 100–150, 150–200, and above 200% of the winning finishing time for each sex and year respectively. The finishing time analysis served as an estimation of physical fitness and a proxy for the more extreme doses of exercise. Swedish registries (Swedish National Patient Registry for diagnoses and Statistics Sweden for socio-economic data) (Hallmarker et al. 2018) were used for information on the date of birth, sex, and education level. The study cohort (n = 395,369) was followed in the Swedish National Patient Registry (detailed information below) throughout 2010.

### The Swedish National Patient Registry

We used the Swedish National Patient Registry to retrieve psychiatric and somatic diagnoses. It covers 99% of all hospital-based diagnoses and contains all primary and secondary diagnoses in patients attending hospital-based care in Sweden since 1987, including hospital-based out-patient visits since 2001. Primary care diagnoses are not included in this registry. Bipolar disorders were defined according to the International Classification of Diseases (ICD), tenth revision (ICD10), or ninth revision (ICD9). Diagnoses included are (F30, F29, F310, F311, F312, F313, F314, F315, F316, F317,F318, F319, 296A, 29610, 296C, 296D, 296E, 29600, 29610, 29620, 29630, 29688, 29699).

### Statistical analyses

We used R statistical software package, considering P-values < 0.05 statistically significant. Demographic data are presented as the median and interquartile range (IQR) or numbers (n) and percent (%). Mann–Whitney U tests were used to estimate numeric and Pearson’s χ^2^ tests to estimate categorical group differences. The risks of bipolar disorder for skiers vs non-skiers were compared with C_ox_ regression models and presented as hazard ratios (HR) with 95% confidence intervals (CI). As presented in the graph, numbers at risk were derived from survival tables specifying the number of individuals entering each 5 year interval. The years between participation in the ski race (and the same year for the matched non-skier) and event or censoring were used to calculate the time variable. The event was a bipolar disorder. When subjects died or at the time of register outtake, censoring appeared. The Causes of Death Register (CDR), held at the National Board of Health and Welfare, was used for information on the date of death for deceased study individuals. To assess the proportionality assumption, we modeled Schoenfeld residuals graphically. Since sex was suggested to be a possible effect modifier, men and women were also analyzed separately. To ensure a considerable separation between participants with the highest and lowest fitness levels, the finishing time was trichotomized to 100–150%, 150–200%, and above 200% of the winning finishing time for men and women separately, to assess the impact of fitness level. We adjusted for age, sex, and education in the adjusted Cox model. All individuals who got diagnosed with bipolar disorders within five years of inclusion were excluded in primary sensitivity analyses. Since bipolar disorder is often difficult to distinguish from other psychiatric disorders in the initial phases, all individuals diagnosed with any psychiatric disorder (depression, anxiety, schizophrenia, or bipolar disorder, see Additional file 1: Table S2) within 5 years from baseline were excluded in additional sensitivity analyses.

## Results

### Ski race participation is associated with a lower incidence of bipolar disorder

**Fig. 1:**
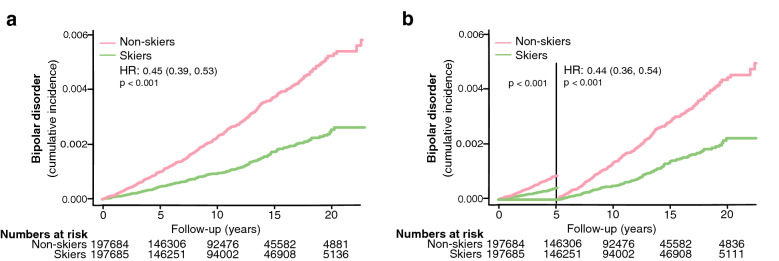
The risk of developing bipolar disorders in skiers compared to non-skiers (**a**) and the risk of developing bipolar disorders more than 5 years after completing the ski race (**b**). HR represents hazard ratios from an unadjusted C_ox_ regression

A total of 395,369 individuals were followed over 3,987,396 person-years. Table 1 displays demographic data comparing the skiers and non-skiers. A total of 715 individuals were newly diagnosed with bipolar disorders after a median follow-up of 10 (IQR 5–15) years. Participation in the long-distance ski race was associated with a lower risk of developing bipolar disorders compared to non-skiers during the follow-up period (unadjusted HR 0.45, 95% CI 0.39–0.53, Table 2, Fig. 1a). Non-skiers had lower education than skiers (Table 1), but adjustments for sex, age, and education did not change the results (adjusted Cox model, Table 2). The effect was not altered even when excluding individuals that developed bipolar disorder within 5 years of the ski race (baseline) (unadjusted HR 0.44, 95% CI 0.36–0.54, Table 2, Fig. 1b). The results remained in additional sensitivity analysis where all individuals developing any psychiatric disorders within 5 years of inclusion were excluded (see Additional file 1: Table S2) and in a sub-analysis including only skiers and non-skiers aged 20–30 years at inclusion [HR 0.44 (0.34, 0.57), p < 0.001, unadjusted Cox]. Taken together, ski race participation was associated with a relative risk reduction of 54% for developing bipolar disorders.

**Table 1:** Characteristics of the study population, presented for the whole cohort and by skiers and non-skiers separately

	All	Skiers	Non-skiers
	*n* = 395,369	*n* = 197,685	*n* = 197,684
Characteristics 1989–2010	Median (IQR) or n (%)	Median (IQR) or n (%)	Median (IQR) or n (%)
Age at baseline, y	36.0 (29.0–46.0)	36.0 (29.0–46.0)	36.0 (29.0–46.0)
Women	149,796 (38)	74,897 (38)	74,899 (38)
Education			
Primary/elementary school (≤ 8 years)	49,344 (13)	14,538 (7.4)	34,806 (18)^a^
Secondary school/high school (9–12 years)	176,571 (45)	76,635 (39)	99,936 (51)
Higher education/university (≥ 13 years)	166,133 (42)	106,147 (54)	59,986 (31)
Bipolar disorder at follow-up.	715	225	490^a^

*IQR* interquartile range, *y* years, *n* numbers

^*a*^*p* < 0·001. Group difference between skiers and non-skiers, estimated with Wilcoxon test (numeric variables) and Pearson’s χ^2^ test (categorical variables). Only significant differences are noted in the table

**Table 2:** Association between physical activity and incident bipolar disorders, based on participation in a long-distance ski race (skiers) compared to non-skiers

Bipolar disorder	Unadjusted model	Adjusted model^a^
Physical activity	HR (95% CI)	HR (95% CI)
*Nr events*	715	703
Non-skiers (Reference)	1	1
Skiers	0.45 (0.39, 0.53)	0.48 (0.41, 0.56)
Excluding bipolar diagnoses < 5 years		
*Nr events*	459	453
Non-skiers (Reference)	1	1
Skiers	0.44 (0.36, 0.54)	0.45 (0.37, 0.55)

C_ox_ regression models showing HR for risk of bipolar disorders

^a^Model adjusted for age, sex, and education

*HR* hazard ratio, *CI* confidence interval

### Both men and women participating in the ski race have a lower incidence of bipolar disorder

**Fig. 2:**
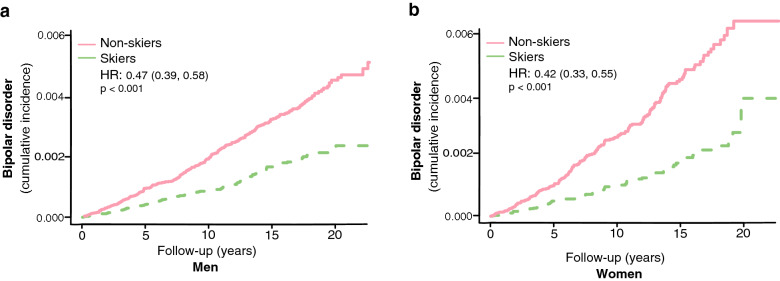
The risk of developing bipolar disorders in skiers compared to non-skiers in men (**a**) and women separately (**b**). HR represents hazard ratios from an unadjusted C_ox_ regression

Both men and women who participated in the ski race had a lower incidence of bipolar disorder (unadjusted HR 0.47, 95% CI, 0.39–0.58 for men and unadjusted HR 0.42, 95% CI, 0.33–0.55 for women, Fig. 2a, b). The results remained significant even if all individuals developing any psychiatric disorders within 5 years of inclusion were excluded (see Additional file 1: Table S3).

### The impact of exercise dose on incident bipolar disorder differs between men and women

**Fig. 3:**
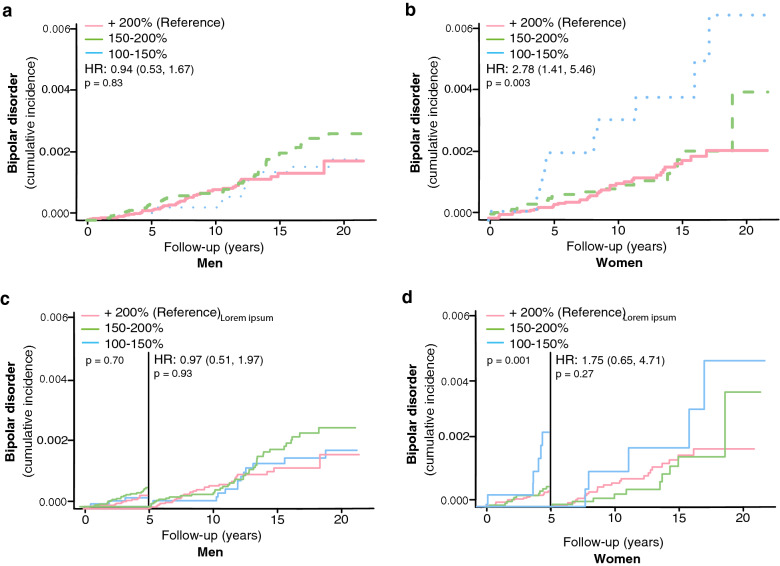
The impact of ski race finishing time on the risk of developing bipolar disorders in skiers in men (**a**) and women separately (**b**). The impact of ski race finishing time on the risk of developing bipolar disorders more than 5 years after completing the ski race in men (**c**) and women (**d**). HR represents hazard ratios from an unadjusted C_ox_ regression for the fastest (100–150% of winning finishing time) group, using + 200% as the reference group

No impact of the ski race finishing time (a proxy for the effect of extreme exercise) on the risk of bipolar disorder was seen among the skiing men (unadjusted HR 0.94, 95% CI, 0.53, 1.67, Table 3, Fig. 3a). Oppositely, we detected a higher risk of developing bipolar disorder among women completing the race with the shortest finishing time compared to slower skiers (unadjusted HR 2.78, 95% CI, 1.41, 5.46, Table 3, Fig. 3b). The results remained even after adjusting for age, sex, and education (adjusted Cox model, Table 3). Nevertheless, when excluding cases diagnosed with bipolar disorder within the first 5 years the association among the women became non-significant (unadjusted HR 1.75, 95% CI, 0.65, 4.71 for women, Table 3, Fig. 3c, d).

**Table 3:** Association between ski race finishing time and incident bipolar disorders in men and women

Bipolar disorder	Men	Women
Finishing time (% of winning time)	HR (95% CI)	HR (95% CI)
Unadjusted model		
> 200% (Reference)	1	1
150–200%	1.31 (0.89, 1.93)	0.96 (0.56, 1.64)
100–150%	0.94 (0.53, 1.67)	2.78 (1.41, 5.46)
Adjusted model^a^		
> 200% (Reference)	1	1
150–200%	1.30 (0.88, 1.92)	0.83 (0.49, 1.43)
100–150%	0.96 (0.54, 1.72)	2.07 (1.03, 4.13)
Excluding bipolar diagnoses < 5 years		
Unadjusted model		
> 200% (Reference)	1	1
150–200%	1.19 (0.75, 1.91)	0.76 (0.36, 1.59)
100–150%	0.97 (0.51, 1.87)	1.75 (0.65, 4.71)
Adjusted model^a^		
> 200% (Reference)	1	1
150–200%	1.19 (0.74, 1.90)	0.64 (0.31, 1.35)
100–150%	1.01 (0.53, 1.94)	1.23 (0.45, 3.37)

C_ox_ regression models showing HR for risk of bipolar disorders in men and women respectively

^a^Model adjusted for age and education

*HR* hazard ratio, *CI* confidence interval

## Discussion

Following almost 400,000 persons, we found that engaging in more physical activity at baseline was associated with a 55% lower risk of developing bipolar disorder during the up to 21 years long follow-up. In addition, we discovered a sex-specific association between the exercise dose (as measured by ski race finishing time, a proxy for fitness level) and the risk of developing bipolar disorder.

Our study contributes with new knowledge in addition to the findings in adult men presented by Åberg et al. (2012), since we also include women in our analyses. Our results show that both men and women participating in the ski race had a 53–58% lower risk to develop bipolar disorder during the up to 21 years of follow-up, indicating that physical activity may be equally beneficial for both sexes. Excluding all persons diagnosed with bipolar disorder within the first 5 years did not alter the results, suggesting that the associations were not significantly driven by reverse causation. Since bipolar disorder is often misdiagnosed for other psychiatric disorders initially and many patients are comorbid with for example anxiety, we also conducted an additional sensitivity analysis excluding all individuals diagnosed with other psychiatric disorders (anxiety, depression, and schizophrenia) during the first 5 years, to reduce the likelihood for reverse causation driven by psychiatric comorbidity and misdiagnosis to influence the results. Our main results were robust even after this extensive exclusion. However, the participant’s average age at baseline was 36. Since bipolar is often developed in early adulthood (before the age of 30), it could be argued that many participants had already passed the most critical period in life with regard to the risk of getting bipolar disorder. Still, our sensitivity analysis confirmed the same association with a lower risk of bipolar disorder among the skiers aged 20–30 years at inclusion.

Interestingly, we found an impact of the finishing time of the ski race on the risk for bipolar disorder among the women, but not among the men. Women completing the ski race faster had about 1.8 times higher risk of getting diagnosed with bipolar disorder compared to slower skiers. A similar pattern was also shown by Åberg et al. even though they only included men in their analysis (Aberg et al. 2012). This non-linearity in the relationship may also be the reason why some study designs, such as the Mendelian randomization, fail to detect a relationship with some types of physical activities (Sun et al. 2020). In fact, the cumulative incidence of bipolar disorder among women in the fastest skiing group reached the same level as those not participating in the ski race. Several studies have suggested that heavy exercise may provoke manic episodes in individuals prone to bipolar disorder (Malhi and Byrow 2016). Nevertheless, when excluding all individuals who developed bipolar disorder during the first 5 years this difference became nonsignificant. Thus, reverse causation seems to be an important driving factor for the association between high physical performance and a higher risk of bipolar disorder among women. This indicates that the higher levels of bipolar disease in the fastest skiing group do not necessarily depend on their dose of physical activity or fitness level, but that women with tendencies to being diagnosed with bipolar disorder are more likely to engage in heavy exercise. Of importance, the eventual hazard of the high-intensity exercise was not possible to investigate with our study design as the follow-up time between the exposure (ski race) and disease onset (diagnosis) is very long, whereas if heavy exercise does facilitate mania, this side-effect would most probably be induced within the next hours to days. Further, our study is of epidemiological nature and does not allow for conclusions regarding the direction of this relationship. Hence, we should be careful with concluding that exercise may trigger mania. Still, monitoring individual changes in physical activity behaviors in patients with bipolar disorder may serve as a strategy to detect early signs of relapse into either a depressive or manic episode (Zebin et al. 2019; Antosik-Wojcinska et al. 2020; Beiwinkel et al. 2016). Of note, the absolute levels of physical activity are strongly person-dependent and the key to this method is to evaluate personal changes over time, where the increased physical activity appears to predict manic relapses and decreased levels predict depressive periods (Grunerbl et al. 2015). Contrarily, several of these studies report both manic and depressive episodes to be predicted by decreased physical activity (Antosik-Wojcinska et al. 2020; Beiwinkel et al. 2016), even though Marco Di Nicola et al. reported exercise addiction to be more common among people with bipolar disorder compared to healthy controls (Di Nicola et al. 2010).

We have not investigated any potential mechanisms behind the possible beneficial effects associated with a physically active lifestyle in our study. Nonetheless, a few previous studies have suggested that beneficial effects may be mediated by stabilizing circadian patterns and improving sleep quality and quantity (Hearing et al. 2016; Dunster et al. 2021; Merikangas et al. 2019; McGlinchey et al. 2014). Indeed, a growing number of studies point out sleep management to be essential for coping with bipolar disorder, not only for diagnosed patients but also for susceptible close relatives at genetic and environmental risk to develop the disorder (Shou et al. 2017; Burgess et al. 2020; Cour Karottki et al. 2020; Vreeker et al. 2021; Firth et al. 2020). Moreover, many patients themselves perceive exercise as something that may bring structure to their chaos (Wright et al. 2012), which may prevent relapses and hospitalization. One should not underestimate the augmented life quality due to the improvements in cardiovascular and metabolic wellbeing as a consequence of a more physically active lifestyle. Going deeper, there is a need to investigate molecular mechanisms inside the brain following exercise in this patient group as not much is known in this area.

Finally, if we aim to use physical activity interventions or monitoring as a preventive strategy to manage the risk of developing bipolar disorder, we need to gain greater knowledge in factors predicting the efficiency of such activities. As sleep management seem to be crucial, the timing of the physical activity may be important. For instance, avoiding too vigorous exercise in the evening may be especially important to optimize the effects as this may aggravate the already existing evening orientation seen among these patients (Dunster et al. 2021). Also, factors linked to adherence and the need for support have to be better defined.

### Strengths and limitations

To the best of our knowledge, this is the first study following such a high number (395,369 individuals) of both sexes for such a long time (up to 21 years) to investigate how a physically active lifestyle affects the risk of developing bipolar disorder. We used the unique population registries available in Sweden. The Swedish national patient registry is one of the largest in the world covering diagnoses set on the entire population since 1964.

Due to the study design, our study has certain limitations as disclosed before (Svensson et al. 2019). In brief, the skiing population smoke less and have a better diet compared to the control population and we are not able to adjust for this. Nor does our study setup takes into account the risk of bias due to family composition, i.e. the tendency for partners to share genetic and life-style-related features such as a lower risk of psychiatric disorders, which may eventually also result in a lower risk for the offspring to develop bipolar disorder and hence also to adhere to a healthy life-style including ski race participation. Medication may influence an individual’s ability to engage in physical activity, but we had no access to data on prescribed medication to use for validation of diagnoses or to adjust or stratify on. Furthermore, it usually takes some time for a patient with bipolar disorder to get the proper diagnosis as a psychotic episode/symptom may be the first clear signs of illness. Therefore, we additionally included unspecified non-organic psychosis (ICD-10, F29) in our analysis, as not to miss any illness in the early phase of bipolar disorder or to overestimate disease-free time after baseline. It may be argued that inclusion of polymorph psychosis should have been included.

Additionally, we have no direct assessment on physical activity levels as we use the participation in the ski race as a proxy for a physically active lifestyle. We also lack data on physical activity among our non-skiers, but previous studies indicate that on a group level, the skiers are significantly more active (Farahmand et al. 2003; Carlsson et al. 2007). Moreover, even if we have an extensive list of exclusion criteria and performed sensitivity analyses to reduce the impact of reverse causation, we were not able to exclude all diagnoses that could prevent ski race participation.

## Conclusions

Taken together, our study followed 395,369 men and women for up to 21 years using diagnoses from the Swedish patient registry. Being a skier was associated with a pronounced risk reduction of bipolar disorder in both men and women. This association persisted even after the exclusion of individuals diagnosed with bipolar disorder or other psychiatric disorders (anxiety, depression, schizophrenia) during the first 5 years. Our results imply that the previously shown preventive potential of physical activity in bipolar disorder in men is valid also among women and that exercise dose may matter. However, as our study groups were not matched on confounding socioeconomic and genetic factors, the detected differences in risk of future bipolar disorder should be interpreted with caution. There is a need for better mechanistic insights as well as elucidation of surrounding predictive factors and the importance of exercise timing for this patient group.

## Supplementary Information

**Additional file 1: Figure S1.** Flow diagram describing the Vasaloppet Study population. **Table S1.** Additional exclusion criteria. **Table S2.** Additional sensitivity analyses. **Table S3.** Additional sensitivity analyses stratified on sex.

## Data Availability

The database for the Vasaloppet cohort with disease incidence belongs to Uppsala Clinical Research Center and can be made available upon request.
