# Supplementary material for: A physically active lifestyle is associated with lower long-term incidence of bipolar disorder in a population-based, large-scale study
Source: Int J Bipolar Disord. 2022 Nov 1;10:26. doi: 10.1186/s40345-022-00272-6 (PMC9622955; doi:10.1186/s40345-022-00272-6)
Supplement: Supplementary file 1 — Additional file 1: Figure S1. Flow diagram describing the Vasaloppet Study population. Table S1. Additional exclusion criteria. Table S2. Additional sensitivity analyses. Table S3. Additional sensitivity analyses stratified on sex. [file 40345_2022_272_MOESM1_ESM.docx]

**Supplementary material**

**1. Extended Method description**

**2. Supplementary Figure**

**3. Supplementary Tables**

**1. Extended Method description**

**Exclusion criteria and matching procedure**

Individuals with severe disease were excluded from the Vasaloppet study since such diseases may hinder participation in a demanding long-distance race. The numbers excluded due to severe disease can be seen in the flow diagram in Supplementary Figure S1. The ICD-codes for these diagnoses have been stated previously(1). In addition, we excluded participants with diagnoses listed in Table S1.

A control individual from the general population was assigned for every ski race, so that skiers participating in Vasaloppet several times got several controls in the first matching process. To get equally many skiers as non-skiers, we performed a re-matching procedure. Since we only used the index race for each skier, the non-skiers would have been older as a group if we had included one control for every time a skier participated in the race.

**2. Supplementary Figure**

**Vasaloppet Study population**

Supplementary Figure S1. Flow diagram describing the Vasaloppet Study population.

**3. Supplementary Tables**

**Supplementary Table 1. Additional exclusion criteria**

| **Diagnosis** | **ICD-9** | **ICD-10** |
| --- | --- | --- |
| Alzheimer´s disease | 331A/3310, 29010 | F00, G30 |
| vascular dementia | 290E, 2904, 2930 | F01 |
| all-cause dementia | 290, F070, 294C, 294B, 331A, 310A, G318A | F00, F01, F02, F03, G30 |
| Lewy body dementia | 331X, G318A, 33182 | F028 |
| dementia in Parkinson disease | 294B, 332A | F023 |
| Parkinson disease | 332A, 3420 | G20 |
| meningitis/encephalitis | 3200, 320A, 320B, 320C, 320D, 320W 320X, 321A, 321B, 321C, 321D, 321E, 321X, 322A, 322B, 322C, 320X, 323, 3230 | G00, G01, G03, G04, G05 |
| epilepsy | 345, 3450 | G40 |
| depressive episode | F399, 296B, 296X, 29620, 29800 | F32, F33, F34, F38 |
| anxiety disorders | 300A, 300B, 300C, 300D, 300D, 3000, 3001, 3002, 3003 | F40, F41, F42 |
| bipolar disorder | 296A, 29610 ,296C, 296D, 296E, 29600, 29610, 29620, 29630, 29688, 29699 | F30, F29, F310, F311, F312, F313, F314 ,F315, F316, F317, F318, F319 |
| schizophrenia | 295, 297, 2970, 2979, 29999 | F20, F21, F22, F23, F24, F25, F28, F29 |
| mental disorders due to the use of alcohol | 291, 2910, 2919 | F10 |

**Supplementary Table 2. Additional sensitivity analyses**

| **Bipolar disorders** | ***Unadjusted model*** | ***Adjusted model**** |
| --- | --- | --- |
| **Excluding psychiatric diagnoses** <5 years** |  |  |
| ***Nr events*** | 408 | 402 |
| Non-skiers (Reference) | 1 | 1 |
| Skiers | 0.47 (0.38-0.58) | 0.48 (0.39-0.60) |

HR: hazard ratio, CI: confidence interval

Cox regression models showing HR for risk of bipolar disorders.

*Model adjusted for age, sex, and education.

** Excluding all individuals who developed any psychiatric disorders (depression, anxiety, schizophrenia, or bipolar disorder, see Supplementary Table 1) within five years of inclusion.

**Supplementary Table 3. Additional sensitivity analyses stratified on sex**

| **Bipolar disorders** | ***Men*** | ***Women*** |
| --- | --- | --- |
| **Excluding psychiatric diagnoses** <5 years** |  |  |
| ***Unadjusted model*** |  |  |
| *Nr events (non-skiers, skiers)* | 169, 86 | 107, 46 |
| HR Non-skiers (Reference) | 1 | 1 |
| HR Skiers | 0.50 (0.38-0.64) | 0.42 (0.30-0.60) |
| ***Adjusted model**** |  |  |
| *Nr events (non-skiers, skiers)* | 164, 86 | 106, 46 |
| HR Non-skiers (Reference) | 1 | 1 |
| HR Skiers | 0.49 (0.38-0.65) | 0.48 (0.33-0.68) |

HR: hazard ratio, CI: confidence interval

Cox regression models showing HR for risk of bipolar disorders.

*Model adjusted for age, sex, and education.

** Excluding all individuals who developed any psychiatric disorders (depression, anxiety, schizophrenia, or bipolar disorder, see Supplementary Table 1) within five years of inclusion.

**References**

1. Hallmarker U, Michaelsson K, Arnlov J, Hellberg D, Lagerqvist B, Lindback J, et al. Risk of recurrent ischaemic events after myocardial infarction in long-distance ski race participants. Eur J Prev Cardiol. 2016;23(3):282-90.
